# Social Waves in Giant Honeybees Repel Hornets

**DOI:** 10.1371/journal.pone.0003141

**Published:** 2008-09-10

**Authors:** Gerald Kastberger, Evelyn Schmelzer, Ilse Kranner

**Affiliations:** 1 Institute of Zoology, University of Graz, Graz, Austria; 2 Seed Conservation Department, Royal Botanic Gardens, Kew, West Sussex, United Kingdom; Max-Planck-Institut fuer Neurobiologie, Germany

## Abstract

Giant honeybees (*Apis dorsata*) nest in the open and have evolved a plethora of defence behaviors. Against predatory wasps, including hornets, they display highly coordinated Mexican wave-like cascades termed ‘shimmering’. Shimmering starts at distinct spots on the nest surface and then spreads across the nest within a split second whereby hundreds of individual bees flip their abdomens upwards. However, so far it is not known whether prey and predator interact and if shimmering has anti-predatory significance. This article reports on the complex spatial and temporal patterns of interaction between Giant honeybee and hornet exemplified in 450 filmed episodes of two *A. dorsata* colonies and hornets (*Vespa* sp.). Detailed frame-by-frame analysis showed that shimmering elicits an avoidance response from the hornets showing a strong temporal correlation with the time course of shimmering. In turn, the strength and the rate of the bees' shimmering are modulated by the hornets' flight speed and proximity. The findings suggest that shimmering creates a ‘shelter zone’ of around 50 cm that prevents predatory wasps from foraging bees directly from the nest surface. Thus shimmering appears to be a key defence strategy that supports the Giant honeybees' open-nesting life-style.

## Introduction

Giant honeybees (*Apis dorsata* and *A. laboriosa*) belong to the oldest honeybee species after the dwarf honeybees (e.g. *A. florea*), having evolved about five to ten million years ago [1], [2]. Unlike the more recent cave-dwelling honeybees (e.g. *A. cerana*, *A. mellifera*), Giant honeybees build their nests predominantly in the open, suspending their roughly semicircular combs from overhead supports such as tree branches, rocks or buildings [for general biology of Giant honeybees see 2]–[12]. In the open, they are directly exposed to a variety of predators, particularly to birds and wasps [3], [8], [13]. This predatory pressure apparently gave rise to the evolution of a series of defence strategies [2], [5], [6], [13], [14], [15].

Generally, defence behaviors of honeybees may involve physical contact with aggressors. A prominent example is their capacity to recruit stinging guards [15] and to mobilize a whole army of defenders. In Giant honeybees, mass mobilization of stinging guards may occur within a split second, and gave them the reputation of being the most dangerous stinging insects on earth [6], [8], [13], [16]. Against wasps, which are major predators of bees, honeybees have developed specific defence behaviors such as heat balling of wasps that come into direct contact with the honeybee nest. Heat balling has been reported for *A. cerana*, *A. mellifera* [17], [18] and *A. dorsata* [19], [20]. For this purpose, the bees heat their thoraces by their flight muscles to above 45°C, a temperature that is lethal to wasps.

Honeybee colonies also defend themselves without physical contact with their enemies, minimizing the risk for the defending bees. Examples include the aposomatic coloration, which is characteristic of all hymenopterans [21], the colony aggregation [2], [13], [22], and shimmering behavior [5], [6], [8], [14], [16], [23], [24]. Shimmering has been observed in *A. cerana* and *A. florea* [2], and in *A. dorsata*, it is a notable visual cue that is impressive even to humans [3], [8]. Shimmering involves an intriguing capacity for very rapid communication within the nest (movie S1), in which hundreds of individual bees flip their abdomens upwards in a split second forming Mexican wave-like patterns [25]. These wave-like figures are ineffective to stop larger predators such as birds [13] or mammals from feeding directly from the Giant honeybees' nests. Field observations [14] suggest that shimmering is provoked especially by wasps. However, the evolutionary role of shimmering and, in particular, its significance as a defence behavior are so far only hypothesized [for summary see 2], and the precise relationship between Giant honeybees and potential predators regarding shimmering is not understood.

This article investigates whether Giant honeybees succeed defending their nests against hornets by shimmering, and how prey and predator interact. The honeybee colony behavior is analyzed concerning the occurrence, strength and repetitiveness of shimmering under the aspects of proximity and velocity of predatory wasps. This proves shimmering as a colony response to approaching wasps. On the other hand, the wasp behavior has been investigated in response of the time course and the strength of shimmering regarding proximity to the honeybee nest. In two different scenarios of experiments, shimmering waves, in particular in their ‘big-scale’ shape, are proved to repel wasps within a specified range around the honeybee nest. ‘Small-scale’ shimmering is effective in preventing wasps from predation by generating ‘confusion’. It is demonstrated that the predation activity of wasps near honeybee nests and the defence responses of Giant honeybees through shimmering base on a reciprocal, mutually adjusted relationship with possibly coevolutionary roots. Lastly, it is discussed how shimmering benefits Giant honeybees, in particular to support their open-nesting habit.

## Materials and Methods

### Species, study site

This article investigates the prey-predator interactions between colonies of Giant honeybees (*A. dorsata*) and *Vespa sp*. hornets at two water towers in the Agricultural Campus of the Tribhuvan University of Kathmandu, Rampur, Chitwan, Nepal in November 2004. The aim was to analyze the wave-like shimmering behavior [8], [23], [24] of Giant honeybees in response to the predatory hornets that hovered around the bee nests. Two scenarios were chosen at two experimental nests where hornets had regularly been observed. The first experimental nest (*scenario A*) consisted of 8000 bees, measuring one meter in the horizontal span and was located at the external rim of the water tower. The colony had arrived just one day before observations had started. It formed a cluster with a small central comb. The second test colony (*scenario B*) was slightly smaller than the first one; it had also migrated to the ceiling of the same water tower some days before experimentation and was also a cluster without a central comb.

### Scenarios investigated

In both scenarios, a single camera recorded the behaviors of hornets and bees in PAL format enabling a frame-by-frame analysis at a rate of 25 images per second. Prior to these experiments documented in this article, the authors had observed thousands of shimmering waves in hundreds of Giant honeybee colonies on several expeditions in India and Nepal over 15 years. Based on this broad experience, both scenarios of the two experimental nests were recognized to typify the shimmering behavior of giant honeybees and the respective flight behaviors of hornets under the given prey–predator relations.

**Figure 1:**
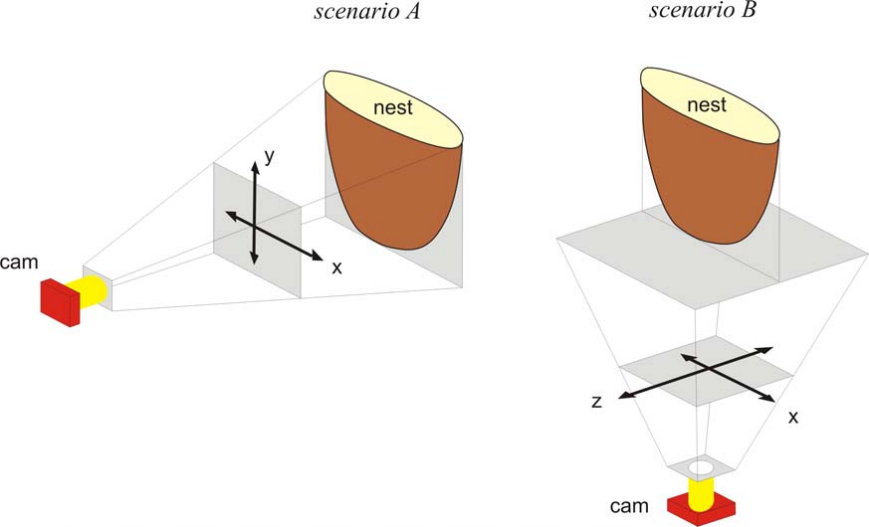
The two different perspectives used in the experimental *scenarios A* and *B*. The nest-specific axes were defined by their real-world coordinates: x, the horizontal (sideways) directions in regard to the vertical flat of the *nest*; y, the up- and downward directions; z, towards and off wards the nest as projected on the horizontal plane. The camera (*cam*) imaged the x-y projection in *scenario A* and the x-z projection in *scenario B*. Note, the distances of the hovering wasp from the bee nest (d_xz_) were addressed in *scenario B* as projection on the x-z plane.

In *scenario A*, predatory hornets were observed hovering around the honeybee nest regularly provoking shimmering behaviors (126 shimmering waves in 77 s). For reference, we analyzed the nest situation before the hornets had appeared (354 shimmering waves in 168 s). The camera obtained views of the front flat of the nest (Fig. 1). The flight trajectories of the hornet were, therefore, documented in the projection of the horizontal and vertical nest-specific real-world coordinates, that is, to the left and the right side of the nest (defined as x-dimensions), and upwards and downwards of the nest (defined as y-dimensions).

In *scenario B*, 203 shimmering waves were observed in the presence of two predatory hornets; 88 cases referred to a single hornet, 115 cases to two hornets that were simultaneously around the nest. In total, 318 episodes of hornets were traced under shimmering activity. The camera documented the scenery from the bottom view (Fig. 1), which referred to the horizontal (x-dimension) real-world coordinates and to the z-dimensions, which are defined as the directions ‘toward and off ward’ the flat expansion of the honeybee nest.

### Assessment of honeybee colony behaviors

**Figure 2:**
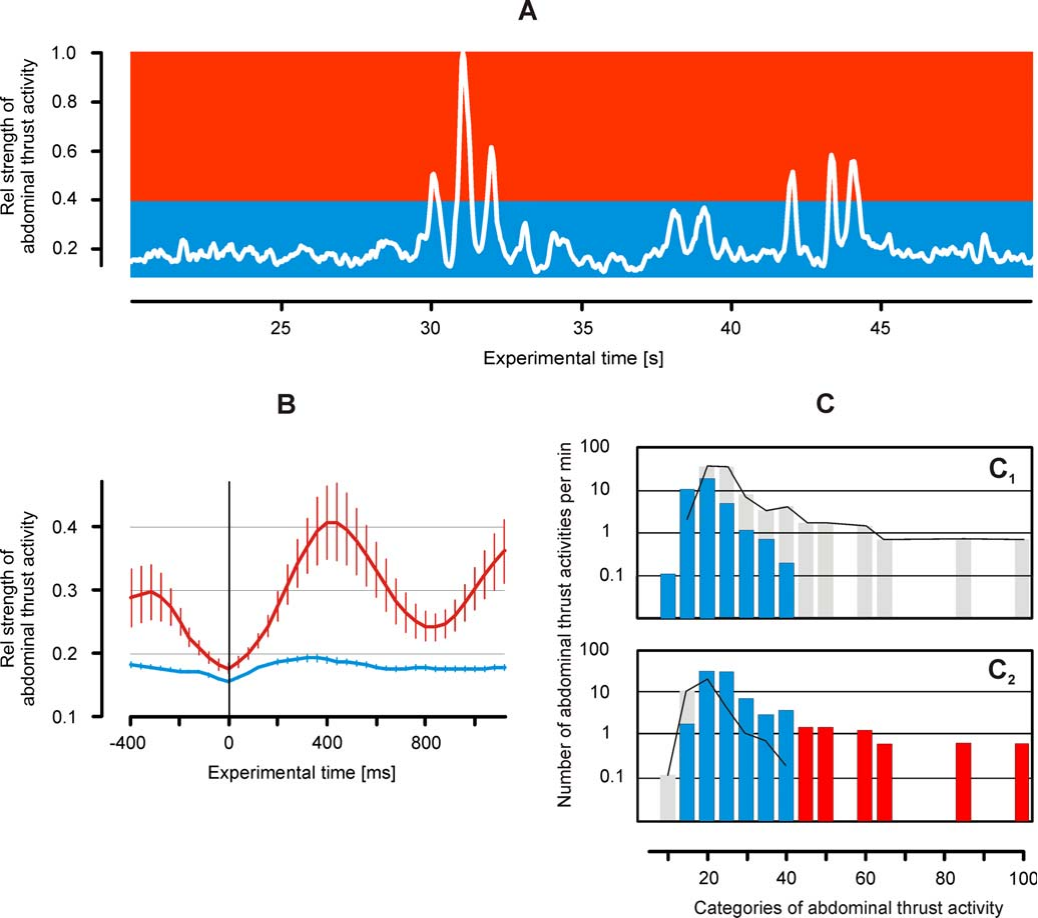
(A) Continuous assessment of abdominal thrust activity of the experimental Giant honeybee nest, while a predatory wasp was present in front of it (*scenario A*); the ordinate value gives the relative strength of thrust activity, the maximum value (1.0) refers to the maximum strength of shimmering (max W_peak_) as observed during 300 s after the onset of waves; the threshold at rW = 0.4 discerns small-scale (blue area) from big-scale (red area) waves; (B) the time course of big-scale (red curve) and small-scale (blue curve) waves; curves show arithmetical means of the respective waves, thin vertical lines denote SEMs; abscissa, rel experimental time, time zero is defined by the onset of the abdominal thrust activity (corresponding to waving); (C) the rate of abdominal thrust activity per min of the experimental nest in two behavioral contexts, (C_1_) ‘undisturbed by a hornet’ and (C_2_) ‘disturbed by a hornet’; abscissa gives the categories of abdominal thrust activity as percentage of maximal waving strength; the blue columns refer to small-scale waves, the grey columns and the thin lines in the background give the respective rates of the opposite behavioral context for comparison. The distributions of the rates of abdominal thrust activities differed between C_1_ and C_2_ insofar that the proportions of the occurrences of waves regarding both states varied from one category to the other (Chi-square test, P<0.001, f = 49). Furthermore, under the presence of wasps the abdominal thrust activities show generally higher rates (Wilcoxon Signed Rank Test: P = 0.017).

**Figure 3:**
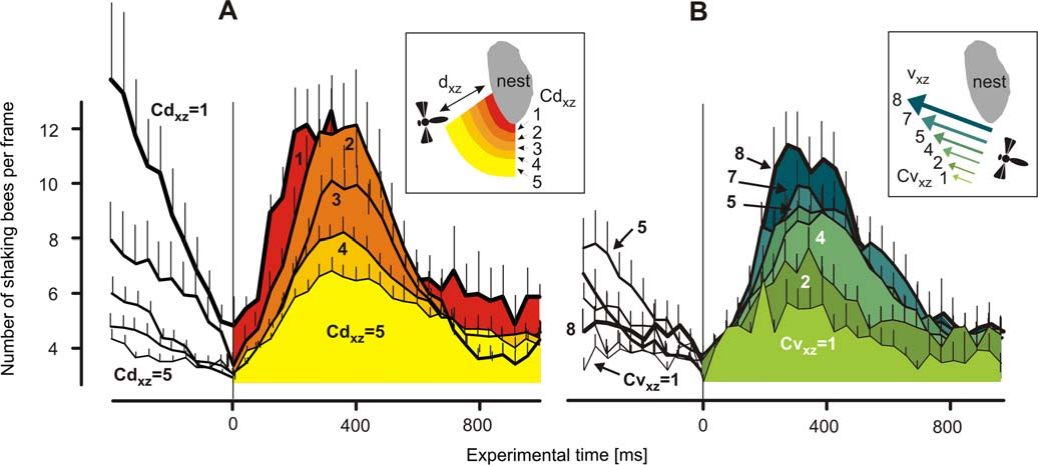
The time courses of shimmering of Giant honeybees in response to approaching hornets (*scenario B*). The waving strength W ( = number of abdomen-shaking bees per frame) depends on the hornets' distances from the nest d_xz_ (A) and on the hornets' flight velocities v_xz_ (B); time zero defines the onset of the waves; (A) five d_xz_ classes (Cd_xz_ = 1–5; coded in yellow to red; for definition, see Methods and Fig. 4,5) and (B) eight v_xz_ classes (Cv_xz_ = 1–8; coded in green to blue) of hornet flight episodes were considered; d_xz_ and v_xz_ class values were assessed from hornets in the 400 ms interval prior to the start of shimmering. Curves show arithmetical means, thin vertical lines denote SEM. For data details, see table 1 and 2.

The focus of this article is on shimmering behaviors. Shimmering is made up by abdominal movements of quiescent individuals predominantly at the surface of the honeybee nest, displaying wave-like processes. These abdominal movements of surface bees were detected by image analysis (Image-Pro, Flir), assessing the shimmering waving strength (*W*) frame by frame. Shimmering was detected in three steps, which were controlled by automated and manual decisions. In a first step, the time course of movement activities was traced. The criterion for automated detection of a peak in movement activity was that the W-value of the reference frame had to exceed the W-values three frames before and after the threshold value, which was defined at 0.9% of the overall maximum of shimmering. Second, the time of the onset of this movement activity was traced by searching for the minimum value of this waving session, 3–10 frames backwards from the peak time. Here, the maximum period of 10 frames considers the time course of shimmering that exhibits a maximum of less than 400 ms (Figs. 2,3). As the third step, the nature of shimmering was identified as a wave-like process. Here, manual control was needed to distinguish shimmering from other movement activities, such locomotor activities as walking, dancing or flying.

**Table 1:** The modulation of shimmering by hovering hornets regarding their distance to the honeybee nest (*scenario B*).

Categories of distance of the hornets from the nest before shimmering		Number of episodes	Distance of the hornets from the nest before shimmering	Shimmering activity as response to the presence of the hornets	
Cd_xz_	d_xz_ [cm]	n	d_xz_ [cm]	W_peak_	W_600_
1	0–18	6	12.7±1.8	12.6±1.3	71.1±4.4
2	18–30	19	25.9±0.7	12.1±2.0	67.1±6.9
3	30–40	49	35.8±0.4	10.0±0.8	55.2±2.9
4	40–60	79	49.2±0.7	8.2±0.5	49.4±2.0
5	>60	48	71.5±1.5	6.5±0.5	42.4±2.1
		201			

Shimmering activities of 201 episodes in terms of W_peak_ and W_600_ in dependence of the pre-wave distances of the hornets from the nest (definitions, see Methods). The hornet data (d_xz_) were assessed in the interval 400 ms prior to shimmering. Shimmering responses (means±SEM) were categorized by five classes (Cd_xz_ = 1 to 5) of d_xz_ (cf. Fig. 3A).

**Table 2:** The modulation of shimmering by hovering hornets regarding their flight velocities (*scenario B*).

Categories of flight velocity of the hornets before shimmering		Number of episodes	Distance of the hornets from the nest before shimmering	Shimmering activity as response to the presence of the hornets	
Cv_xz_	v_xz_ [cm s^−1^]	n	d_xz_ [cm]	W_peak_	W_600_
1	10–20	7	41.15±6.04	6.43±0.72	37.08±2.63
2	20–25	25	40.27±3.33	7.49±0.75	43.23±3.51
3	25–30	29	41.01±3.38	9.28±0.98	51.36±3.42
4	30–40	53	38.60±2.05	8.88±0.90	53.08±3.23
5	40–50	30	47.23±3.53	9.06±0.83	55.58±3.97
6	50–60	21	47.68±4.71	8.86±1.04	53.36±5.24
7	60–80	21	53.78±4.01	9.89±0.95	58.28±4.20
8	>80	16	56.98±5.35	11.14±1.26	61.88±5.58
		202			

Shimmering activities of 202 episodes in terms of W_peak_ and W_600_ in dependence of the hornets' pre-wave flight velocities. The hornet data (v_xz_) were assessed in the interval 400 ms prior to shimmering. Shimmering responses (means±SEM) were categorized by eight classes (Cv_xz_ = 1 to 8) of flight velocity v_xz_ of the hornets (cf. Fig. 3B).

The wave strength W was originally defined and calibrated as the number of surface bees per frame that were actively ‘shaking’ or ‘lifting’ their abdomens. This ‘wave’ parameter was also applied in a more general sense as one of the ongoing aspects of movement of nest mates, regardless of whether or not shimmering had been identified as the respective process. For explanation, the value W_peak_ characterized the intensity of shimmering at its peak, and the value W_−400 ms_ was used to quantify the levels of movement activity 400 ms before the onset of the shimmering waves (which may include residual waving activity).

To estimate the number of bees that participated in a shimmering wave, the integral ∫*W(t)* was calculated and this value was calibrated by a correction factor *f*_corr_. Calibration needed the manual counting of the real number of bees that had lifted their abdomen in the referenced images. This calibration procedure was necessary because a single abdominal shaking lasts for 160 ms [14] and in shimmering, the movement detection by image analysis is particularly sensitive to the onset of shaking. As a representative estimate for the shimmering strength, the number of bees participating in the first 600 ms after the start of shimmering was defined by the formula W_600_ = ∫*W(600 ms)/f*_corr_.

### Assessment of hornet behaviors

The *scenarios A* and *B* provided two different perspectives (Fig. 1) regarding the honeybee nest and referred to different sets of flight parameters of the predatory hornets. Using image analysis (Image-Pro, Flir), behavioral parameters was assessed frame by frame (in steps of 40 ms) in both scenarios. The positional coordinates of the hornet with regard to the x-y plane in *scenario A* and with regard to the x-z plane in *scenario B* were measured. In *scenario A*, the movement components (Δx/40 ms, Δy/40 ms) of the hornets in front of the bee nest were measured and the respective angular parameters of flight direction (α_xy_/40 ms), turning behavior (θ_xy_/40 ms), and the nondirectional parameter of flight velocity (v_xy_) were calculated. In *scenario B*, the shortest distance of the hornet to the nest surface (*d*_xz_) and its differentiation over time that defines the distance velocity *v*_dxz_ = Δ*d*_xz_/Δt were determined. The turning angle per time θ_xz_/40 ms documented changes in flight direction in the x-z projection, which was calculated as the difference between the flight directions (α_xz_ {1,2}, α_xz_ {2,3}) displayed in two successive pairs of frames ({1,2},{2,3}). Here, the hornets displayed *scenario B* (nest-)-specific behaviors; they approached the nest predominantly from one direction, that is, from the bottom right side of the image. In the image-based standard, the hornets in *scenario B* turned counter-clockwise ‘away from the nest’ after shimmering, and turned clockwise ‘toward the nest’. ‘Turning away from the nest’ was coded as positive and ‘turning toward the nest’ as negative, to get the same signs for the shimmering of the bees and the turning behaviors of the hornets in the comparing graphs.

The flight velocity *v*_f_ was calculated by v_f_ = ds/dt (with ds as length of the flight path per time interval dt) in the respective projection planes (v_f_ = v_xy_ for *scenario A*, and v_f_ = v_xz_ for *scenario B*). The v_f_ value in its time course is a good indicator for changes in the hornets' flight behavior near the honeybee nest, if the focus is on the reactivity of the hornets to the shimmering waves. But in *scenario B*, its absolute levels were also considered.

### Analysis of interactions/distinguishing ‘action’ and ‘reaction’

The behaviors of both predator and prey have been synchronized to the onset of the shimmering waves. This trigger concept not only associates the shimmering waves of the bee colony to the hornet behaviors considering shimmering as responses to the hornets' behaviors, but also vice versa, that is, it allows considering hornet behaviors as responses to shimmering. The shimmering waves and the flight behaviors of the hornets were monitored from 400 ms prior to until 1000 ms after the onset of shimmering. The question was whether the flight behavior of the hornet in the vicinity of the honeybee nest represented adequate cues for affecting shimmering. In detail, the investigation was on whether the strength of the shimmering waves (W) was dependent on the flight parameters of the hornet (d_xz_, v_dxz_, v_f_) in the pre-wave period, 400 ms before the onset of waving. To the contrary, changes of the hornet behavior in the first 600–1000 ms after the onset of shimmering was tested as obvious responses to shimmering.

### Categorization of bee-hornet episodes

An episode between the honeybee as prey and the hornet as predator was defined by the shimmering behavior of the bee colony and by the flight behaviors of the hornet in the front of the bee nest. As detailed in earlier sections, the *scenarios A* and *B* refer to different geometrical perspectives and therefore, in particular, to different sets of flight parameters of the hornets. Besides that basic difference, both scenarios enabled to provide distinct concepts of evaluation. In *scenario A*, ‘big-scale’ and ‘small-scale’ waving activities were distinguished according to their peak strength, which was above or below the threshold of 40% of the maximum wave strength W that was monitored in the total experimental session. Big-scale processes represented shimmering waves that spread over the nest surface, affecting hundreds of bees to lift their abdomens sequentially. Small-scale processes were local, wave-like reactions of small groups of abdomen-thrusting bees and have not exceeded the number of 10 active bees.

**Figure 4 pone-0003141-g004:**
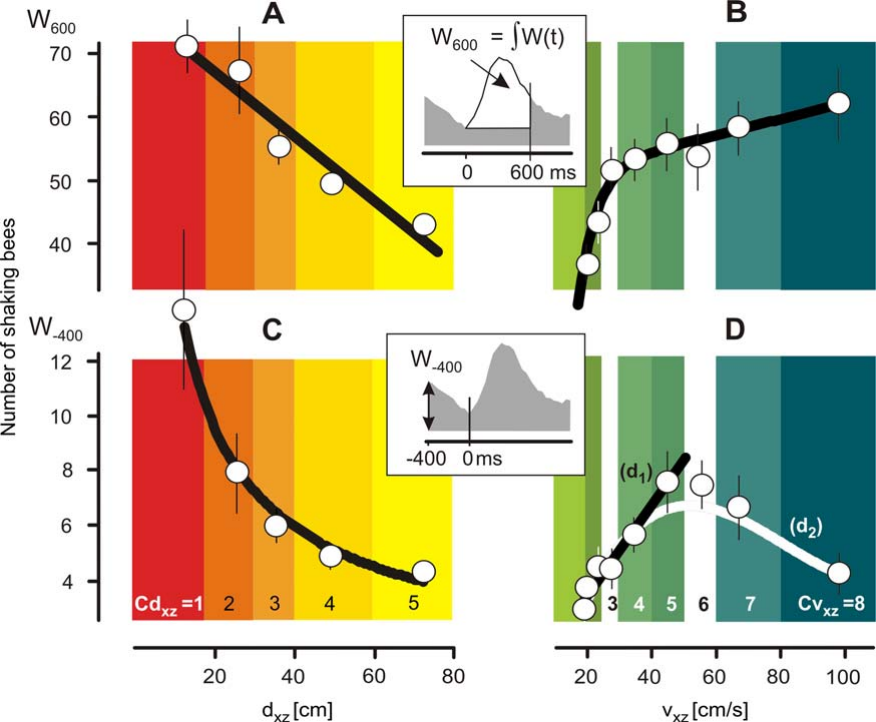
The effect of flight behavior of predatory hornets on the waving strength of Giant honeybees (*scenario B*). The waving strength W_600_ gives the numbers of bees which had shaken their abdomens over 15 frames (definition see inset and text); it depends on the hornet's distance from the nest d_xz_ (A) and on the hornet's flight velocity v_xz_ (B); time zero in the insets defines the start of the shimmering waves (cf. Fig. 3); (A,C) red to yellow shaded areas define the five d_xz_ classes, and (B,D) green to blue shaded areas define the eight v_xz_ classes of hornet flights as used in Fig. 3 (for definition see Methods); open circles are arithmetical means, thin vertical and horizontal lines denote SEM; thick lines are regressions of the mean values regarding to 201 wave episodes with 317 flight episodes of two wasps (Cd_xz_ = 1 to 5; Cv_xz_ = 1 to 8); regression A: r = −0.969, P = 0.006; regression B: r = −0.963, P = 0.015. W_−400_, the amplitude of the waving strength 400 ms before the onset of the consecutive wave (see inset and text for definition), giving the residual shimmering strength under repetitive conditions; W_−400_ declines with d_xz_ (regression C: r = −0.989; P = 0.022), but inclines regarding v_xz_ at lower velocity levels (Cv_xz_ = 1–5: regression d_1_: r = 0.975, P = 0.005) and shows an overall nonlinear relation for Cv_xz_ = 1–8 (regression d_2_: r = 0.857; P = 0.027). All tests refer to Polynomial Regression (SigmaStat).

In *scenario B*, the episodes were categorized in two other ways. First, by the distance d_xz_ of the hornets to the honeybee nest prior to the onset of shimmering. For that, the d_xz_ values in the 400 ms before shimmering was averaged and the episodes were sorted into five classes of pre-wave distances (Cd_xz_ = 1 to 5, table 1), whereas the class widths were chosen according to the statistical incidence of the hornet episodes. If two hornets were present, the d_xz_ value of that hornet that was nearer to the nest was used to categorize the shimmering wave. The second method of categorization considered the flight velocities (v_f_ = v_xz_) of the hornets in the same 10 frames prior to shimmering. Eight classes of pre-wave flight velocity (Cv_xz_ = 1 to 8, table 2, see Results, chapter ‘Shimmering as response to predatory hornets’) of the hornets were defined. If two hornets (w_1_,w_2_) were present, a combined v_w1+w2_ value was calculated using a linear correction model considering the particular d_xz_ value for each hornet (dw_1_;d_w2_): v_w1_+_w2_ = v_w1_*f_w1_+v_w2_*f_w2_ with f_w1_ = 1−(d_w1_/(d_w1_+d_w2_)) and f_w2_ = 1−f_w1_. This procedure weighted the nearer hornet more than the distant one because of the linear relationship between hornet proximity and waving strength (Fig. 4A).

### Categorization of ‘reactive’ and ‘nonreactive’ hornets

In *scenario B*, the shimmering waves and the flight behaviors of the hornet were categorized by the following two principles: The waving episodes were judged by manual decision based on gross inspection of honeybee and hornet behaviors, whether or not the hornet had the chance to provoke shimmering, and whether or not the hornet was in the position to be affected by the wave. In the video, these behavioral categories were clearly distinguishable (see the ‘reactive’ hornet demonstrated in movie S2,S3). If hornets responded to a wave, independently whether they provoked it or not, the respective episodes (n = 149) and the hornets were termed ‘reactive’. In close vicinity to the nest (d_xz_<15 cm) all episodes were ‘reactive’.

Shimmering is not necessarily a response to wasps, it may also be provoked by homing and departing bees (Fig. 2). It is intricate to judge whether hornets hovering near the honeybee nest are or are not in the position to respond to a shimmering wave. If they would be not, the episodes and the hornets were termed ‘nonreactive’ (n = 167). Three typical examples may illustrate the conditions for the categorization of ‘nonreactive’ episodes: First, if a hornet hovered near the nest but was relatively too far away from the nest (e.g. more than one meter away) and additionally, too slow (e.g. when hovering at the spot). In this case, the hornet was not necessarily disposed to elicit shimmering, and it was also not likely to respond to shimmering provoked by any other sources. Second, if a hornet chased a homing bee the hornet's body length axis was usually directed to the fleeing bee, and mostly away from the honeybee nest. Under this condition, the hornet hardly showed any sign of response to the shimmering wave, which might have been elicited by itself or by other sources. Third, if one hornet interacted with another hornet in front of the giant honeybee nest, both of them were concentrated on each other and were obviously not able to respond to the shimmering which they might have provoked.

While the category of ‘reactive’ hornets is unequivocally defined, the category of ‘nonreactive’ hornets remained to be crucial because of its dependence on gross subjective manual criterions, which did not allow deciding in detail about any residual pattern of reactivity of the hornets to e shimmering. To proof whether ‘nonreactive’ hornets did not respond to shimmering or showed any odd reaction patterns, we tested the time courses of the respective behaviors of hornets categorized as ‘nonreactive’ for the existence of any obscure responsiveness.

### Assessment of ‘direction fidelity’ of predatory hornets

In *scenario A*, the flight trajectories of the predatory hornets in the x-y projection were observed and the data during 400 ms before and 1200 ms after the onset of the shimmering wave considered. The mean trajectories of the hornet flight were compiled by integrating the mean positional changes (*m*Δx, *m*Δy) regarding two 40 ms time intervals relative to the onset of shimmering, and the relevant mean distances (*m*D_xy_ = (*m*Δx^2^+*m*Δy^2^)^0.5^) per 40 ms were calculated. The value *m*D_xy_ factually is the result of vector subtraction, because they refer to data of pooled individual flight paths, and therefore *m*D_xy_ is a function of turning ranges ρ_xy_ of the hornet (ρ_xy_ = max θ_xy_−min θ_xy_) rather than a function of the flight velocities v_xy_ of the hornet (compare Results, chapter ‘Small-scale and big-scale shimmering’). Assuming that the hornet has, per 40 ms, a maximum turning range of ρ_xy_ = 180°, the scalar values *m*D_xy_ of the mean heading *m*θ_xy_ follow the equation *m*D_xy_ = cos ρ_xy_. In terms of statistics, *m*D_xy_ is shorter the more the turning angles θ_xy_ of the hornet deviate from one frame to the other. Therefore, *m*D_xy_ is a useful measure for the range of deviations in the directional flight behavior of the hornet. Calibrated between 0 and 1 (rel *m*D_xy_ = a* *m*D_xy_) for the turning ranges from 180° to 0°, rel *m*D_xy_ describes the ‘direction fidelity’ of the predatory hornet. In other words, if the hornet keeps its flight directions constant from one frame to the other (that is with a turning range of ρ_xy_ = 0°), it performs a high level of direction fidelity, which is reflected by the value rel *m*D_xy_ = 1. To the contrary, if the hornet turns by θ_xy_ = ±90° within 40 ms, the turning range is maximal (ρ_xy_ = 180°), and the level of direction fidelity is minimal (rel *m*D_xy_ = 0).

### Statistics

Gaussian distributed data sequences were compared by parametric tests (t-test). If the normality test failed, the software automatically used nonparametric tests (Chi-square test, Wilcoxon Signed Rank Test). These tests traced differences in behaviors between two experimental states, e.g. how the behaviors of the hornets differ between two time intervals, such as before and after the onset of shimmering. Correlations were characterized by the regressions of the original data values of the respective behavioral classes or facultatively (see below) of their arithmetic means. The regressions were fitted by optimizing their coefficients of determination (R^2^) and tested by Spearman rank order correlation test.

The time courses of the behaviors of honeybees and hornets in three steps were compared, using the One Way Repeated Measures ANOVA (e.g. Friedman test on Ranks, Sigmastat). The time correlations of the original data over a number of discrete time intervals in 40 ms steps were proved (e.g. from time zero to 400 ms after the onset of waving) and was adjusted for ties. In the first step, a test was conducted to see whether the pairs of variables of prey (W) and of predator (v_dxz_, θ_xz_) tend to increase together during the experimental time after the onset of shimmering in two time intervals (0–400 ms; 400–800 ms). Here, Dunns Method tested the original data per time interval, and the Spearman test their mean values. In a second step, the relative differences among the treatment groups of prey (rel W) and of predator (rel v_dxz_, rel θ_xz_) were proved. In a third step, if the relative differences were because of random sampling variability, the respective regression of the arithmetical means of the same time intervals as the appropriate description of the correlation was accepted. This three-step statistical procedure estimated the type of interaction between prey and predator, although the original predator data would have been too unfriendly for a straight one-way Repeated Measures ANOVA.

## Results

### Occurrence of shimmering

In this study, the conditions under which shimmering waves are produced by giant honeybee colonies were investigated. In *scenario A*, the waving activities at the surface of the experimental honeybee nest under two conditions were monitored, (a) when a wasp was hovering in front of the nest (observation time: 77 s; Fig. 2, movie S4), and for reference (b) without a wasp around the honeybee nest (observation time: 168 s). These abdominal movement activities (see Methods) were categorized into small-scale (n = 72) and big-scale (n = 37) waves. In small-scale activities, only tens of bees raised their abdomens. The wave strength in the pre-wave period is low because the respective signals are nonrepetitive; its time course (blue curve in Fig. 2B) peaked after 200–250 ms. Big-scale waves (red curve in Fig. 2B) spread over the nest, reached their maximum activity typically after 400 ms and were repetitive. Consequently, the pooled data in the pre-wave phase of the reference wave exhibit residual traces of the preceding wave cycle.

Wave-like processes also occur without the presence of a hovering wasp. Foraging nest mates departing from or arriving at the nest mostly are the source for the generation of shimmering in absence of predators. However, in *scenario A*, big-scale waves only occurred under the presence of predatory wasps (Fig. 2C, red columns). The rates of wave-like processes differed between the states ‘with wasp’ and ‘without wasp’ (Chi-square test, P<0.001, f = 49); here, the proportions of the occurrences of waves varied regarding both states from one wave strength category to the other. Furthermore, under the presence of wasps the wavelike processes showed generally higher rates (Wilcoxon Signed Rank Test: P = 0.017). This finding proved wasps evoked shimmering in close vicinity of the nest.

### Shimmering as response to predatory hornets

#### Waving strength

This study investigates several flight parameters of predatory hornets in the vicinity of Giant honeybee colonies regarding their significance for eliciting shimmering. In *scenario B*, shimmering behavior was measured by its waving strength W and it was categorized by two parameters of the hornets' flights in the pre-wave phase. The first parameter was the proximity d_xz_ of the hornets in respect to the honeybee nest. Here, five classes (Cd_xz_ = 1–5; Fig. 3A, table 1) were considered. The supplemental regression in Fig. 4A summarizes the overall dependency of the waving strength (W_600_) from the proximity of the hornets (d_xz_). It was found that when the hornets were immediately prior to the onset of the wave close to the nest, the strength of the provoked wave was maximal, with a participation of 70 bees in the course of 600 ms (Cd_xz_ = 1, Fig. 4A). Farther away, the hornets caused much smaller waves and aroused fewer bees.

The second parameter to categorize the shimmering responses in *scenario B* was the flight velocity vf = v_xz_. For that, eight classes (Cv_xz_ = 1–8; Fig. 3B, table 2) were considered. Hornets with a speed of less than 20 cm s^−1^ provoked only a weak single wave (Cv_xz_ = 1 in Fig. 3B). A more detailed view shows that less than 40 bees were involved (Cv_xz_ = 1 in Fig. 4B) in the average that corresponded to approximately 60% of the maximal shimmering response. If the hornets flew slightly faster, for example, with more than 30 cm s^−1^, the colony response was much stronger (Cv_xz_ = 4 in Fig. 4B) at more than 80% of the maximal waving strength, which the fastest flying hornets had evoked (Cv_xz_ = 8 in Fig. 4B). This finding demonstrates that wasps elicit strong waves if they fly at moderate speed, but if they hover at the spot the wasps are able to ‘creep’ nearer to the bees without evoking big-scale waves.

#### Repetitiveness

**Figure 5 pone-0003141-g005:**
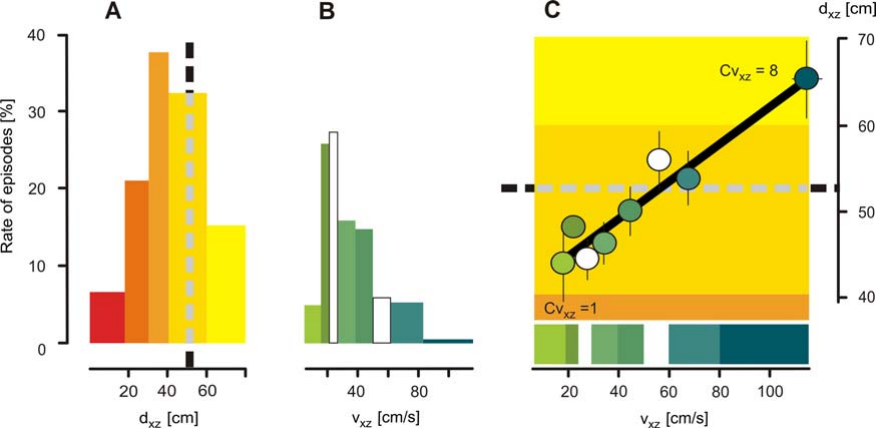
Effect of flight behavior of predatory hornets on the waving strength of Giant honeybees (*scenario B*). Percentage of hornet episodes (n = 317) observed in the respective five d_xz_ classes (A) and eight v_xz_ classes (B) of hornets' flights; (C) the relationship between the flight velocities v_xz_ (abscissa) of individual hornets and their distances d_xz_ to the nest (ordinate); dashed lines (A,C) give the average hovering distance of the hornets (cf. Fig. 6). Regression of the means in C: d_xz_ = 0.215*v_xz_+40.596; Cv_xz_ = 1 to 8; r = 0.962; 317 episodes; P<0.001); for definition and color coding, see Figs. 3,4 and Methods.

The arousal state of a Giant honeybee colony when predatory wasps are nearby is also expressed by the repetitiveness of shimmering. A good measure of it is the residual waving strength W_−400_ (lower inset of Fig. 4), assessed 400 ms before the start of the particular wave. The amplitude of the residual wave strength also depended on both flight parameters, d_xz_ and v_f_ of the predatory hornet (Fig. 4C,D). The repetitiveness of waving increased exponentially the nearer the hornet came to the nest (regression in Fig. 4C). Regarding flight velocity, the repetitiveness of shimmering is seemingly complex and follows an optimum distribution (Fig. 4D). Hornets in the lower range of flight categories (Cv_xz_ = 1 to 5) provoked a linear increase of repetitiveness in shimmering with their flight speed, but in the higher range of flight categories (Cv_xz_ = 6 to 8) the repetitiveness of shimmering decreased with increasing v_xz_ (regression *d* _1_ in Fig. 4D). The overall relation between the repetitiveness of shimmering and the flight speed v_xz_ of predatory hornets is nonlinear (regression *d* _2_ in Fig. 4D). This complex relation results from the fact that v_xz_ itself is a function of the hornet's proximity d_xz_. The auxiliary regression in Fig. 5C showed that the individual v_xz_ correlated with the d_xz_ data of the hornets in the average, slower hornets flew closer at the nest than faster ones. Furthermore, most of the hornets flew within the mean hovering distance of d_xz_ = 52 cm (dashed line in Fig. 5A,C) and slower than 50 cm s^−1^ (Fig. 5B) when they provoked shimmering.

Finally, these data of *scenario B* (Fig. 3,4) make clear why shimmering waves were less repetitive although the hornets were fast. The reason was that fast wasps flew mostly farther away from the nest (Fig. 5C), and their effect on the honeybees to elicit shimmering was consequently less. In other words, repetitiveness of shimmering is primarily a function of proximity.

### Hornet behavior in response to shimmering

Long-lasting experiences over more than one decade with Giant honeybee colonies have enabled the authors hypothesize that shimmering waves do have antipredatory goals. If so, it should be possible to observe that shimmering lowers the chances of the hornets to prey on the curtain bees on the surface of the Giant honeybee nests. In the following sections, this surmise is investigated and questioned whether shimmering is able to distract wasps from grabbing bees, whether it is able to repel wasps or is even able to make wasps turn away from the nest.

#### Shimmering drives hornets away from the nest

**Figure 6 pone-0003141-g006:**
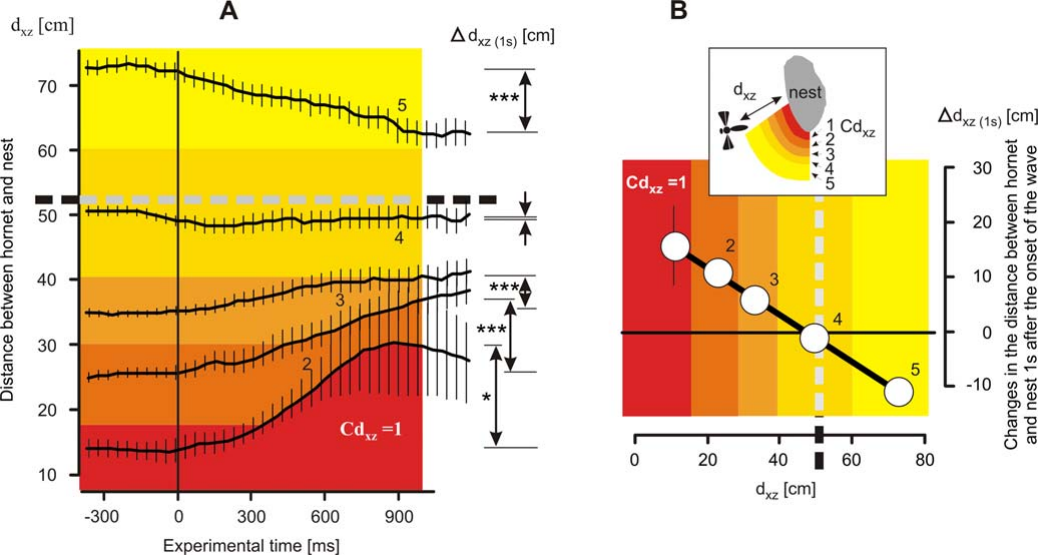
The hornets' responses to shimmering depend on the distance from the bee nest d_xz_ (*scenario B*). Hornet behaviors were categorized in five distance classes Cd_xz_ = 1–5 (see inset, Figs. 3–5 and Methods). Thick lines connect the arithmetical means, thin vertical lines denote SEM;. (A) Wasp behavior monitored for 1600 ms, starting 400 ms prior time 0, the onset of shimmering; Δd_xz (1s)_ values give the changes in the position of the hornet regarding its distance to the honeybee nest within 1 s after the onset of the wave (significance levels: *, P<0.05; **, P<0.01; ***, P<0.001; t-test); the horizontal dashed line, the average hovering distance (d_hov_ = 52.10±0.53 cm; n = 9757 images). (B) Correlation between Δd_xz (1s)_ and d_xz_, the thick line gives the regression of means (Δd_xz (1s)_ = −0.449* d_xz_+22.078; r = 0.998; Cd_xz_ = 1–5; P<0.001; 326 episodes); positive values of Δd_xz (1s)_ at Cd_xz_ = 1–3 represent movements of the hornets away from the bee nest and indicate avoidance responses; the response shown for Cd_xz_ = 4 is neutral, and the negative values of Δd_xz (1s)_ at Cd_xz_ = 5 outside dhov illustrate that the hornets usually approached the nest when shimmering started.

The primary question for an obvious antipredator impact of shimmering on wasps is whether wasps respond to shimmering. In *scenario B*, the hornets hovered in the average at a distance of d_hov_ = d_xz_ = 52.1±0.53 cm (mean±SE; 9757 images; dashed lines in Figs. 5,6) in front of the nest. At this *mean hovering distance*, hornets elicited only weak waves (cf. Cd_xz_ = 4 in Fig. 3A); but if the hornets were nearer than d_hov_ they not only elicited bigger waves, they also withdrew from the nest after the start of shimmering. They increased their distance from the nest more than they were before the wave had started (Cd_xz_ = 1–3 in Fig. 6). However, when they were outside the mean hovering distance (Cd_xz_ = 5 in Fig. 6), they came significantly nearer to the nest as soon shimmering had started.

To quantify this responsiveness of the wasp to shimmering, the change in distance of the hornet within one second after the onset of waving (Δd_xz(1s)_) was chosen. Taken together the responses at all five proximity categories (Cd_xz_ = 1 to 5) the data correlated linearly with the proximity of the hornets to the nest prior to the shimmering wave (regression in Fig. 6B).

Summarizing, the authors conclude that at distances greater than the mean hovering distance, hornets tended to approach the nest under the influence of shimmering. This suggests first, that hornets were attracted by shimmering if they were further away from the nest by at least half a meter. Second, at the mean hovering distance, hornets were not affected by shimmering; they stayed neutral with regard to approaching or leaving the nest site. Third, when the hornets were closer than the mean hovering distance, they withdrew from the nest in the course of shimmering. This hornet behavior is indicative of avoidance behavior, suggesting that shimmering plays a role in repelling predatory hornets, but only when close to the nest.

#### Shimmering makes ‘reactive’ wasps turn away from the nest

**Figure 7 pone-0003141-g007:**
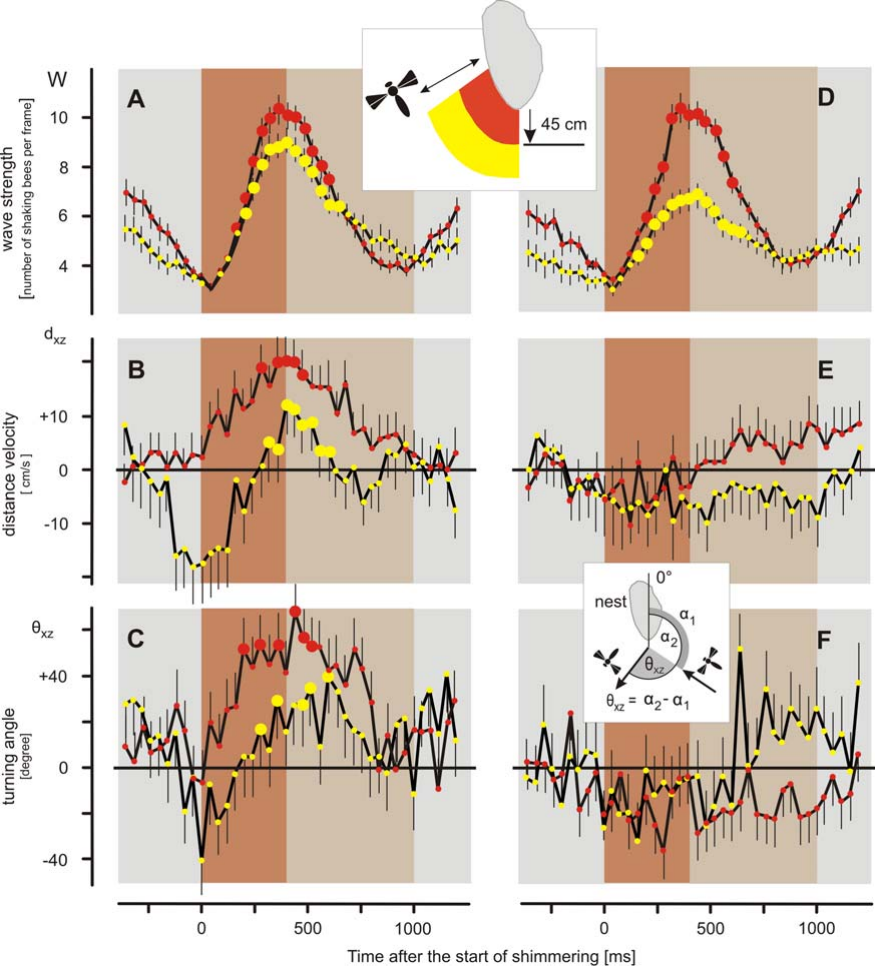
The hornets' behaviors during shimmering (*scenario B*). The time courses of shimmering (A,D) and of the hornets' flights (B–C,E–F), ‘reactive’ (A–C) and ‘non-reactive’ (D–F) episodes (for definition, see text). Honeybee and hornet behaviors were synchronized to the start of the shimmering waves (abscissas give the time in milliseconds after the start of the shimmering waves). The hornets' behaviors are shown in terms of distance velocity vd_xz_ (B,E) and turning angle θ_xz_ (C,F); see inset and text for definition. Two classes of hornets were defined according to their distance to the nest in the 400 ms interval prior to shimmering: d_xz_<45 cm (red circles, 84 ‘reactive’ episodes; 59 ‘non-reactive’ episodes), and d_xz_>45 cm (yellow circles, 65 ‘reactive’ episodes; 108 ‘non-reactive’ episodes). Different brown-shaded areas define two test intervals in relation to the time course of shimmering (brown-shaded: 0–400 ms, grey-brown shaded: 400–1000 ms). Circles and bars give arithmetical means±SEM. Big full (red or yellow) circles give significant differences of the data in relation to the starting time of the wave at t = 0 ms (P<0.05; Holm-Sidak test, Friedman Repeated Measures ANOVA on Ranks).

In *scenario B*, the time courses of the flight behaviour of ‘reactive’ (see Methods) hornets were analyzed when they show the avoidance response to shimmering (Fig. 7). For that, two groups were distinguished regarding their distance to the nest, that is, when they were (a) nearer than the hovering distance (d_xz_<45 cm) or (b) when they were outside the mean hovering distance (d_xz_>45 cm). The average position d_xz_ of the hornet in the 400 ms interval prior to the start of shimmering was again the criterion for the classification of both, the shimmering waves and the hornets' flights.

‘Reactive’ hornets in close vicinity to the nest (d_xz_<45 cm) showed a strong reaction to shimmering and turned away from the nest with a maximum speed v_dxz_ of around 18 cm s^−1^ (Fig. 7A,B, red symbols) and by a turning angle of θ_xz_∼50° (Fig. 7C, red symbols), showing a strong and immediate avoidance reaction. ‘Reactive’ hornets that were more distant from the nest than d_hov_ (d_xz_>45 cm), usually approached the nest when shimmering had started. This is expressed by the negative v_dxz_ and θ_xz_ values in Fig. 7B,C (yellow symbols), which coincide with the values of the wasp outside the mean hovering distance (Cd_xz_ = 5) in Fig. 6. As expected, 300 ms after the start of shimmering, they turned themselves away from the nest displaying positive v_dxz_ and θ_xz_ values. For comparison, the respective time courses of shimmering under the presence of ‘reactive’ hornets display that the shimmering waves were stronger and more repetitive if the hornets were nearer to the nest (Fig. 7A, red symbols) than further away (Fig. 7A, yellow symbols).

**Figure 8:**
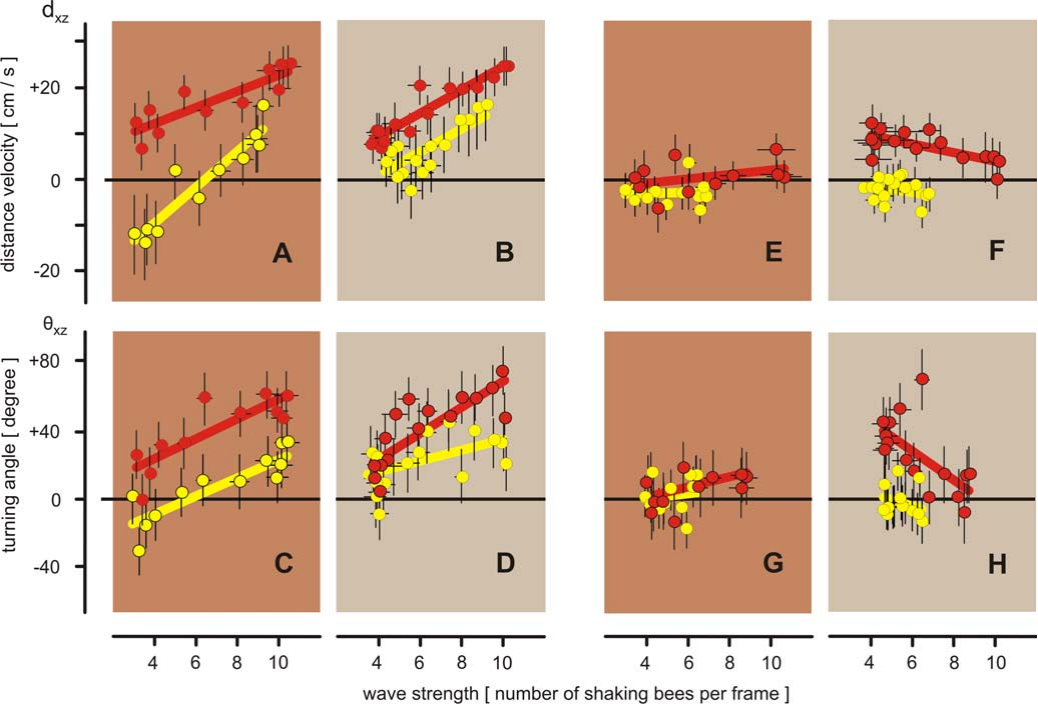
The correlations of the waving strength with the flight behaviors of ‘reactive’ (A–D) and ‘non-reactive’ (E–H) hornets over the time course of shimmering (*scenario B*). Abscissas, the waving strength W assessed by the number of abdomen-shaking bees per frame; ordinates, the hornets' behaviors measured by the parameter distance velocity v_dxz_ (A–B;E–F) and turning angle θ_xz_ (C–D;G–H) using the data of ‘reactive’(A–D) and ‘non-reactive’ (E-H) episodes (cf Fig. 7). Red circles and red lines refer to hornets which were near the nest (d_xz_<45 cm) in the pre-wave period, yellow circles and yellow lines refer to hornets which were further away from the nest (d_xz_>45 cm). Two time intervals were defined in relation to the time course of shimmering (0–400 ms: A,C,E,G; 400–1000 ms: B,D,F,H; for the coding of the brown shaded areas, see Fig. 7). Circles and bars give arithmetical means and their SEM; thick lines give the regression functions (test parameter, see Table S1), which refer to Multiple Linear Regression (Sigmastat) of the arithmetical means (see Methods).

The additional analysis in Fig. 8 shows that the shimmering waves and the behaviors v_dxz_ and θ_xz_ of ‘reactive’ hornets' correlate in both phases of the shimmering waves, that is, in the increasing (Fig. 8A,C) and decreasing part (Fig. 8B,D) of the wave, at least if the means of shimmering and hornets' behaviors were taken in pairs per time interval for correlation (Table S1, see section Methods for the discussion about test strategies). The data suggest that the hornets are not only driven away from the nest by the shimmering wave as documented in Fig. 7, but they are also urged to do so over the entire time course of shimmering. Although the original data give only subsignificant trends, the correlations in Fig. 8 based on the time-interval related means allow assuming that the hornets increase their avoidance behavior in the course of 400 ms, as long as the shimmering wave also steps up in strength. In contrast to ‘reactive’ hornets, ‘nonreactive’ hornets did not show any residual responsiveness to shimmering (Fig. 7D–F, 8E–H).

#### Small-scale and big-scale shimmering

**Figure 9:**
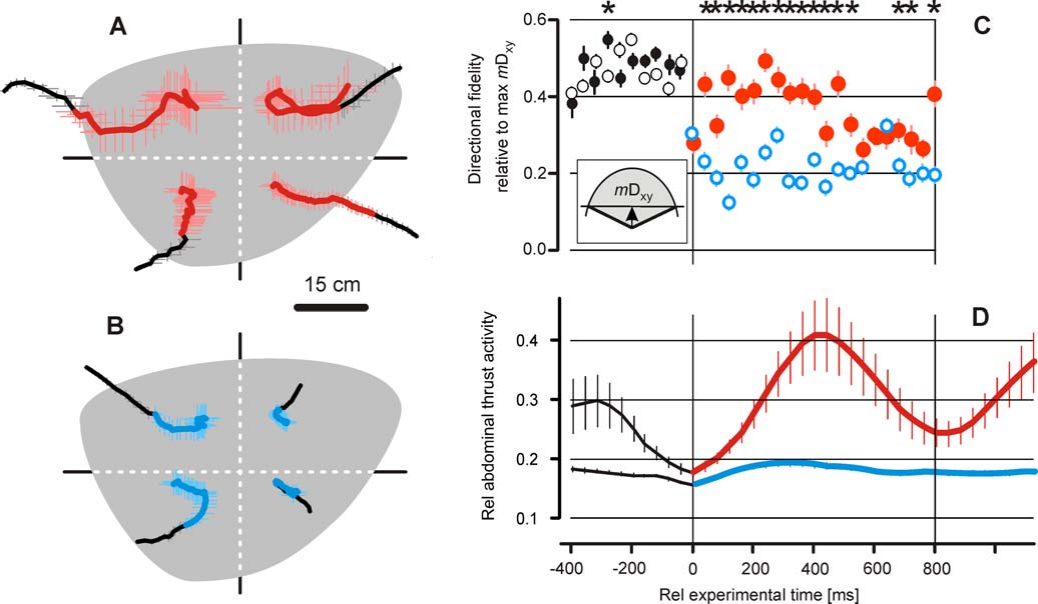
‘Confusion’ of hornets hovering in front of the experimental honeybee nest as observed in big-scale (red color code) and small-scale (blue color code) episodes in *scenario A*. (A,B) Mean trajectories of the approaching hornet (for compilation of trajectories, see text); the sectors between the dashed lines define the four divisions of the mean pre-wave flight directions of the hornets for pooling the x- and y-values of the positions of the hornets approaching to the nest in 40 ms intervals. Thick lines, arithmetical means of x- and y-values of the hornet's position; horizontal and vertical bars, SEM. Note, that the trajectories before the onset of the waves (coded by black thick lines) are straighter than after the onset of the waves (coded by red and blue thick lines). (C) Hornet flight behavior under the influence of big-scale (full red circles) and small-scale (open blue circles) shimmering waves; ordinate, *m*D_xy_ (see inset for definition of the mean vector length *m*D_xy_; grey segment, the defined turning range), calibrated between 0 and 1, the resulting scalar rel *m*D_xy_ is a measure of direction fidelity of the hovering wasp (circles and vertical bars, arithmetical means±SEM). The data show that the direction fidelity of predatory hornets is lowered by shimmering waves, by small-scale waves stronger than by big-scale waves; stars refer to significant (P<0.05, one-way ANOVA test) differences between the responses of the hornet to big-scale (n = 20) and small-scale (n = 33) waves per time interval. (D) The mean time courses of big-scale (red line) and small-scale (blue line) waves (cf. Fig. 2); ordinate, relative abdominal thrust activity (mean±SEM); abszissa, the relative experimental time (C,D) at time zero occurred the onset of shimmering; pre-wave sessions are coded by gray or black, shimmering sessions are coded by blue or red (A–D).

In *scenario A*, the flight trajectories of the predatory wasps were sorted according to two classes of arousal conditions for the hovering wasps, in particular to small-scale and big-scale shimmering (Fig. 2,9). To illustrate the hornet's responsiveness to both levels of shimmering in more statistical way, the trajectories of the hornets were synchronized to the onset of shimmering, and their positional x- and y-data pooled in 40 ms intervals. Four nest sectors (dashed lines in Fig. 9A,B) allowed defining four directional classes of flight trajectories according to the mean positions of the wasp in the 400 ms prior to the onset of the wave (Fig. 9A,B).

*Small-scale shimmering lowered the direction fidelity of the predatory hornet.* The shimmering waves strongly influenced the flight trajectories of the wasp in both, small-scale and big-scale conditions (Fig. 9). During shimmering, the resulting flight paths of the hornet were generally shorter as compared to the pre-wave phase. The reason was not that the hornet would have decelerated its flights, but its turning tendencies deviated much stronger, so that its direction fidelity dropped after the onset of shimmering, which is documented in the x-y plane of *scenario A* (Fig. 9C, blue symbols). Without any directional preference, the hornet turned away from those bees, which it had obviously decided before to prey on. To the contrary, here, big-scale shimmering had less effect on the hornet (Fig. 9C, red symbols).

**Figure 10:**
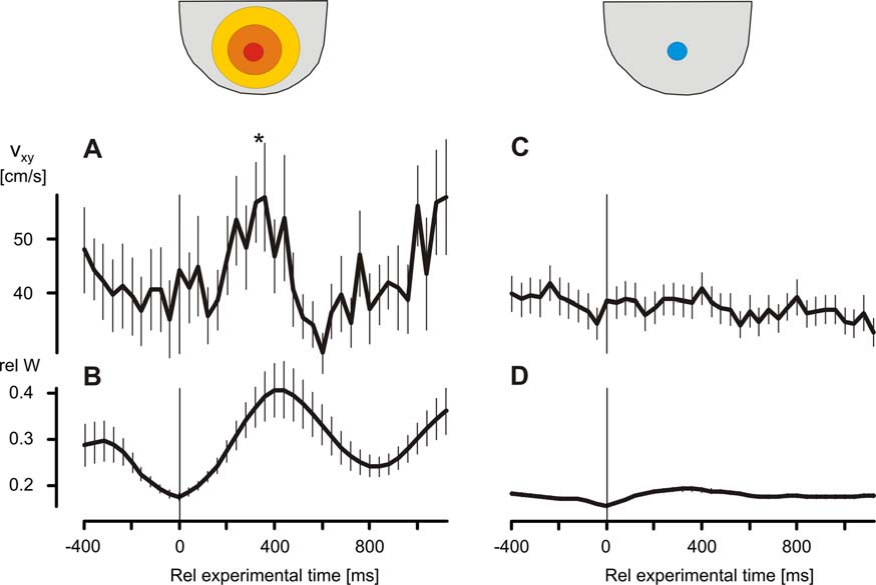
Hornets' responses regarding the flight velocity v_xy_ to big-scale (A,B) and small-scale (C,D) shimmering waves (*scenario A*). Big-scale and small-scale waves were categorized according to a threshold of 40% of the maximal waving strength (cf. Fig. 2). The peak in flight velocity v_xy_ of big-scale wave episodes (A) from 240 to 360 ms after the onset of wave significantly differs from pre-wave v_xy_ values (*, P<0.05, One-way Repeated Measures ANOVA; 15 episodes). Small-scale waves (C,D) obviously do not affect the flight speed v_xy_ of the hornet. Note, that the acceleration pulse of the hornet (A) coincides with the time courses of big-scale waves (B). Abscissa, experimental time in ms; time zero defines the start of shimmering; lines connect arithmetical means, vertical bars give SEM. The sketches above the graphs symbolize big-scale waves (red-orange areas) as spreading over the nest, while small-scale waves (blue area) remain local processes.

*Big-scale shimmering drove the hornet to accelerate.* Under the influence of big-scale waves, the hornet in *scenario A* speeded up, small-scale waves had practically no effect in this respect (Fig. 10). The acceleration pulses terminated after about 400 ms, at the same time when the shimmering waves decreased in amplitude. In other words, big-scale shimmering waves irritate wasps, which speed up for some hundreds of milliseconds. Consequently, these acceleration pulses drive the wasps away from the targets region to which the wasps had directed their predation flights to prey on.

## Discussion

The data obtained from this study provide new insights into the complex spatial and temporal patterns of interaction between bee-hawking hornets and Giant honeybees under defence. In support of their open-nesting life-style, Giant honeybees have evolved a set of defence strategies that keep predatory animals, birds, and wasps in particular, off the nest. The obviously most spectacular defence action refers to the recruitment and release of flying defenders [2], [8], [13], [19], which chase vertebrate disturbers or predators away from the nest by counter-attacking them through their stinging behavior. In addition, honeybee colonies pose an even deadly peril to *Vespine*, *Ropalidiini* and *Polybiini* wasps [26] that touch the nest surface. A group of immediately recruited bees seize such intruders, draw them into the bee curtain and heat-ball them to death [15], [17], [19], [20].

It has been the conventional view [2], [8], [16] that shimmering has been evolved in particular for colony defence in Giant honeybees. It belongs to categories of strategies [27] that pose practically no risk to the defenders. It consumes far less energy than emergency reactions that are released when defence turns to a matter of physical contact with the enemy. This study first presents quantitative proof that shimmering is an anti-predatory response of giant honeybee colonies to the presence of hornets and demonstrated this for both sides of this prey–predator interaction by a series of finely shaped details: The study showed that shimmering waves became stronger and more frequent the nearer a predatory hornet came to the nest and the faster the hornet flew there. In turn, hornets were more affected by shimmering the nearer they came to the honeybee colony. In this intermezzo, substantial evidence was gained that shimmering does have anti-predatory impact on wasps. While local small-scale shimmering may confuse wasps, which had approached the nest into touching reach, big-scale shimmering that may spread over the whole honeybee nest does have the capacity to repel predatory wasps, but only within a restricted limit away from the bee nest.

### Shimmering repels hornets

Bee-hawking hornets incessantly approach honeybee nests, again and again, to prey on them, without showing the tiniest sign of habituation (which was tested in 335 episodes of shimmering waves). The data obtained do not provide any support for the ‘proximity-avoidance’ hypothesis that would propose that hornets are deterred by the honeybee nests and would avoid its vicinity. Nevertheless, the factor ‘proximity to honeybee nest’ apparently modulates their responsiveness to shimmering, essentially because the honeybee colony also alters its defence response depending upon the distance of the intruder.

However, three aspects have been proved in support of the ‘shimmering-repels-wasps’ hypothesis that assumes that bee-hawking hornets show an avoidance response to shimmering when they come too close to the honeybee nest. First, it was proved, particularly in *scenario A*, that shimmering forces the hornets to accelerate their predation flight for some hundreds of milliseconds, which happened just at the peak time of shimmering. This acceleration pulse is sufficiently long to drive the predator away from the region where it had previously intended to prey on. Second, when the hornets were close to the nest, in particular inside the mean hovering distance, the hornets turned off from the honeybee nest as soon as the shimmering wave became sturdy (*scenario B*). Third, the hornets were not only affected by the onset of shimmering, their reactivity correlated with the waving during its whole course. When the number of abdomen-thrusting bees increased, the hornet enforced its avoidance reaction by turning stronger and flying faster away from the nest, and when shimmering declined, the hornet reduced and terminated its shimmering-specific avoidance reactions. However, when the hornets were only slightly further away from the honeybee nest, when shimmering occurred, just outside the zone defined as the ‘mean hovering distance’, this repelling goal of shimmering was reversed. Then, shimmering even attracted the hornets, which were inclined to approach the nest as a region of prey.

### The ultimate goal of shimmering is likely to shelter the nest

Thus, the capacity of shimmering to repel wasps is limited to a distance of around half a meter from the nest, which equals the mean hovering distance. This restriction in the defensive coverage of colony of Giant honeybees is likely to be associated with the obvious ultimate goal of colony defence to generate a safety zone around the nest that should keep predatory wasps away from the nest, preventing them from catching bees directly from the nest surface. If the wasps, nevertheless, succeed in intruding this shelter zone they should not stay long. This is exactly what was observed during shimmering: When wasps approached the nest, the honeybee colony continuously generated shimmering waves that repeatedly repelled the wasp. Shimmering evidently benefits the honeybee colony because it lowers, factually to zero, the hunting success of those predatory wasps that want to seize bees from the nest surface. The chance to observe wasps in trying to seize giant honeybees from the surface of the nests is quite rare. In a total of estimated 30 minutes of own observation over years and of a hundred of trials by the wasp to catch surface bees, we have not observed any successful grasp.

On the other hand, the hornets hardly elicited shimmering when they were more than 50 cm away from the nest. The question here is whether and why giant honeybees do not recognize hornets outside the distance of 50 cm as a threatening peril. It is known that *Apis dorsata* colonies, which had mobilized their guards for a potential counter attack, recognize predatory birds at much greater distances from the nest than the mean hovering distance of wasps. One of the experimental *Apis dorsata* nests in Chitwan instantaneously released hundreds of flying defenders when a kite approached it, although this bird was still far more than twenty meters away. Thus, it seems that Giant honeybee colonies have developed specific distance measures for predatory wasps.

### Visual and pheromone cues of shimmering

Another question associated to the concept of a shelter zone of Giant honey bee nests arises here: does shimmering deliver only visual cues to the wasps or does it also utilize pheromone channels? It is known that shimmering is linked to chemical scenting [14], but there are arguments that make chemical scenting extremely unlikely to trigger the avoidance response of wasps. First, the release of alarm pheromones in honeybees is accompanied by sting protrusion [15], [27]. Stinging activities do not occur during shimmering, but otherwise, alarm pheromones of honeybees do not prevent hornets from hunting bees [2], [17]. Second, shimmering is accompanied by the release of Nasonov pheromone [14]. After a series of repetitious waves, Giant honeybees open their last inter-tergital gaps of their abdomens, exposing the Nasonov glands. Nasonov scent is a social pheromone and signals to the bees to ‘stay together’ [14], thus preventing single bees from changing their roles into those of guard bees (flying defenders) that would fly off to attack the predator. However, there are reasons that make it is impossible for Nasonov pheromone to trigger the avoidance response of an approaching hornet. Firstly, the exposure of Nasonov glands has been only observed after a series of shimmering episodes [14], but hornets were disturbed by shimmering from the first wave onwards. Additionally, and more important is that the latency of the avoidance reaction of the wasp after the onset of shimmering is less than 100 ms, and is therefore by several orders of magnitude faster than the exposure of Nasonov glands and also faster than the obvious spreading of the pheromone would take. Summarizing, the hornet's avoidance behavior appears to be triggered solely by visual cues of shimmering.

### Why social waves against wasps have been evolved?

If wasps should be hindered to feed directly from the honeybee nest it would be sufficient to organize local groups of surface bees to confuse or misguide them [28]–[30]. Bee-hawking wasps should then learn that it is impossible to catch bees directly from the nest, and they will try to find easier prey near the honeybee nest, such as homing or departing worker bees. Any Mexican wave-like synchronisations of hundreds or even thousands of honeybees would then be seemingly too much ado for this defence purpose. Why then have Giant honeybees evolved shimmering that is an extraordinarily complex trait of group defence unique in the whole animal kingdom? The findings of this study allow assuming that the goal of waving must be associated with the open-nesting life style of the Giant honeybees.

As demonstrated earlier (in *scenario A*), local small-scale waves increased the deviation of turning angles of predatory wasps (lowering their direction fidelity) when they hovered close to a Giant honeybee nest (<20 cm). Here, they may well discern single bees, but such local waves make it difficult for the wasps to concentrate upon them. This aspect is termed ‘confusion’, an anti-predatory strategy often cited as an important mechanism in predatory interactions [28], [31]–[33] as the reduced attack-to-kill ratio experienced by a predator resulting from an inability to single out and attack individual prey in a group. However, ‘confusion’ has been proved only for very few predatory interactions [31]. Thus, small-scale waves as a local response of Giant honeybees on the nest surface would suffice to confuse wasps and to prevent predation. To the contrary, big-scale waves were typically provoked by wasps, which were further away from the nest and flew faster. The results show that big-scale shimmering effects much less ‘confusion’ for the hornets, which is plausible because ‘confusion’ of wasps that are further away from the nest would hardly benefit the colony.

Therefore, the authors propose that the wave-like character of shimmering has been evolved obviously not primarily to confuse wasps, but, as shown above, to repel wasps. A possible explanation for this striking capacity probably has two aspects. First, shimmering may reinforce in wasps innate and not habituating fixed action patterns of avoidance. Second, waving possesses a further sophisticated and strikingly ‘convenient’ effect that may also enforce the innate avoidance of the addressee: When the wave of abdomen-thrusting bees spreads over the nest, the wave front stays indeed ‘behind’ the wasp, it factually press-gangs the wasp away from the place it originally wanted to prey on (see movie S4). Subsequently, the wasp is strongly inclined to retreat and fly away from the ‘threatening’ wave front.

### Hunting outside the shelter zone of Giant honeybee nests

Although hornets are continuously attracted to the honeybee nests (by their rich resources of protein and sugar), shimmering effectively prevents the potential predators from collecting bees from the nest surface. Hunting episodes in which hornets continuously attempted to ambush flying bees in front of the honeybee nest were recorded. In thousands of wasp episodes in several honeybee colonies, a single case of successful hunt of a hornet having caught a bee from the nest curtain was not observed. However, hornets do have another kind of hunting success if they focus on ingoing and outgoing bees that cross their hovering range. Bees threatened by the bee-hawking wasp are unprotected by the colony-bound collective defence, but are still able to escape by dodging and fast flight (v_xz_ = 2.25±0.03 ms^−1^, n = 1855 images, n = 107 flights of bees; *scenario B*). Most flying bees escaped the wasps successfully; they either flew off the nest at maximal speed or landed as fast as possible on the nest.

The movies S2,S3 show one example of an unsuccessful trial of the hornet to catch a flying bee. It was attracted by a homing bee; it chased after her, and directed its flight course and its body's length axis exactly toward its target. The bee was totally upset, made an escape round and tried to land as fast as possible. During this manoeuvre, the hornet came closer to the honeybee nest and was repelled by a shimmering wave. This colony response was provoked by the homing bee in union with the hornet. Within 6 min of observation and after 67 trials of hunting, homing or departing bees, the success rate was 3% (corresponding to two bees), which was not a big loss for the bee colony, but still a benefit for the hornets.

### The significance of evolving shimmering in the course of evolution

Theoretically, there is a fundamental problem for a prey–predator relation if a defence action of a potential prey, such as shimmering, does not lead to any physical contact with the enemy. Of course, such traits are less risky for the defenders, but they are obviously less dangerous for the predators, which may learn to ignore ‘unperilous’ signals of the potential prey. However, observations clearly demonstrate that repetitious shimmering efficaciously repels the same hornet again and again. Any habituation effect in hornets can be excluded; obviously, they cannot ignore shimmering, although they repeatedly try, without showing any sign of habituation, to hunt their prey from the honeybee nest. Although the wasps decelerate and approach the nest, shimmering interrupts their landing operations, and elicits avoidance reactions, which take the wasps away from the spot of prey. Mostly they are repelled off the nest, at least half a meter or more, from where they start the next hunting episode.

Because of their persisting and nonhabituating bee-hawking quirks, it is assumed that wasps envisage honeybee nests as a prey of extraordinary attractiveness. Obviously to avoid widespread wasp predation, honeybees have acquired cavity-nesting abilities (in Southeast Asia: *Apis cerana*, *A. nuluensis*; in Eurasia and Africa: *Apis mellifera*). In particular, *Apis cerana* and A. *nuluensis* have strong defence lines against bee-hawking wasps and at their nest entrance they also exhibit shimmering against wasps, although at a far lower level than Giant honeybees [30], [34]. To the contrary, the European honeybee (*Apis mellifera*) has acquired far less effective abilities to thwart the predation of wasps. This can be demonstrated in direct comparison with *A. cerana* [30], because the European honeybee has been introduced from Europe to South East Asia, and it fails under the strange conditions of widespread wasp predation in Southeast Asia. This particularly illuminates a lack of adaptation in the predator–prey relationship to defend against non-European wasps. Therefore, the co-evolution between *Apis cerana* and their autochthon bee-hawking wasp predators must have been very intense [17], [18], [30].

Hence, it has been extremely important for the open-nesting Giant honeybees during their five million years [1] of coexistence with their wasp predators to evolve defence traits that effectively support their life-style. In this article, it is proved that the first defence line of the giant honeybees against wasp includes shimmering behavior. The reciprocal interactions between Giant honeybees and hornets, during their trials to catch bees from the nest and during the subsequent shimmering of the honeybees, are far more complex than mere stimulus–response behaviors would allow to expect. It seems extremely unlikely that the finely shaped, mutually adjusted behaviors (shown in Figs. 3–8) have developed by chance as a kind of general response flexibility. In particular, in view of the observation of experiments with *Apis cerana* and *A. mellifera* specimens in the same apiary [30], these mutual responses between Giant honeybees and wasps are suggestive of co-evolutionary adaptation in a predator–prey relationship. The Giant honeybees as potential prey, have acquired the ability to continuously signal to their wasp predators through shimmering ‘keep distance and do not expect a free meal’. The visual cue of shimmering may thus have a combined impact of signaling vigilance of the prey and of an unprofitability [35] of exploiting the honeybee nest. However, the capacity of shimmering goes beyond this goal, as shimmering is proved to actively repel hornets to prevent predation.

## Supporting Information

Table S1 Regressions of the correlations between shimmering behavior and the hornets' behaviors (see Fig. 7).
(0.04 MB DOC)

Movie S1 This video shows two 130 cm-wide nests of the Asian Giant Honeybee *Apis dorsata* attached to a thick branch of a tree in Assam. The nest in the foreground displays shimmering: a Mexican-wave-like, spiral or circular, pattern. The ‘mouth’ zone of the nest is at the left bottom rim, where forager bees depart, arrive and dance. In contrast, the bees in the periphery are quiescent, but in response to hornet attacks, they produce shimmering (QuickTime; 7.8 MB).
(7.94 MB MOV)

Movie S2 This video shows a nest attached to the ceiling of a water tower in Chitwan, Nepal. A typical, unsuccessful, hunting episode is shown in which a hornet chases a flying bee, but the bee escapes and lands on the nest. The landing of the bee and the manoeuvre of the hornet provokes shimmering, which makes the hornet turn off the nest. The film documents this in original speed (QuickTime; 1.6 MB).
(1.59 MB MOV)

Movie S3 This video shows the same scene as ‘Movie 2’, but in slow motion and contains explanatory text and arrows and the trajectories of the hornet and the flying bee. Light green represents the flying bee, and the shimmering (shaking) nest bees are shown in dark green. The hunting hornet is indicated in red, and in violet when repelled by shimmering. Dark green arrows point out that shimmering repels the hornet. The violet arrow shows the new flight course of the hornet in response to shimmering (QuickTime; 0.9 MB).
(0.90 MB MOV)

Movie S4 This video shows the nest of *scenario A* under the presence of a wasp. The movement activity of the bees from one frame to the other, assessed by image analysis, were displayed as white areas. The red spot marks the thorax of the hovering wasp. Shimmering waves are repetitively produced in different strengths. The yellow line at the bottom traces this shimmering strength by the sum of the white areas; the numbers on the right bottom side give time of observation in seconds. Note, how the wasp is driven under the influence of the shimmering wave to the upper rim of the nest after 6s (QuickTime; 3.2 MB).
(3.27 MB MPG)
