# Supplementary material for: Social Waves in Giant Honeybees Repel Hornets
Source: PLoS One. 2008 Sep 10;3(9):e3141. doi: 10.1371/journal.pone.0003141 (PMC2528003; doi:10.1371/journal.pone.0003141)
Supplement: Table S1 — Regressions of the correlations between shimmering behavior and the hornets' behaviors (see Fig. 7). (0.04 MB DOC) [file pone.0003141.s001.doc]

Table S1. Regressions of the correlations between shimmering behavior and the hornets’ behaviors (see Fig.7).

| **Behaviour**  **category** | **Distance from the nest** | **Interval**  **in ms** | Distance velocity v_dn_ | | | | Turning angle θ | | | |  |
| --- | --- | --- | --- | --- | --- | --- | --- | --- | --- | --- | --- |
|  |  |  | **a**_0_ | **a**_1_ | **R**^2^ | | **a**_0_ | **a**_1_ | **R**^2^ | | **N** |
| **non-reactive** | **d**_n_**<45** | **0-400** | -6,6040 | 0,4571 | 0,1332 | -25,1950 | | 1,9632 | 0,3019 | | 59 |
|  |  | **400-1000** | 9,5243 | -0,8993 | 0,4741 | | 44,9380 | -5,4978 | 0,4083 | | 59 |
|  | **d**_n_ **>45** | **0-400** | -7,5559 | 0,1429 | 0,0063 | | -20,0320 | 0,9468 | 0,0167 | | 108 |
|  |  | **400-1000** | -8,2848 | -1,6227 | 0,0352 | | -8,2848 | -1,6227 | 0,0352 | | 108 |
| **reactive** | **d**_n_ **<45** | **0-400** | 1,1750 | 1,7385 | 0,7605 | | -12,6750 | 5,5684 | 0,6986 | | 84 |
|  |  | **400-1000** | -4,7970 | 2,5064 | 0,8847 | | -19,8040 | 7,2971 | 0,6423 | 84 | |
|  | **d**_n_ **>45** | **0-400** | -29,1270 | 3,9361 | 0,8869 | | -49,7130 | 5,8738 | 0,7507 | | 65 |
|  |  | **400-1000** | -12,6620 | 2,4340 | 0,5105 | | -12,4780 | 3,2255 | 0,2896 | | 65 |

Regression functions (W = a_0_ + a_1_*v_dn_; W = a_0_ + a_1_*θ) of ‘reactive’ and ‘non-reactive’ hornets (for definition, see text) are shown for two positional conditions of the hornet relative to the bee nest (d_n_ < 45cm, d_n_ > 45cm) and for two intervals relative to the start of shimmering (0-400, 400-1000 ms); N, the number of episodes.
